# Creating Gameful Design in mHealth: A Participatory Co-Design Approach

**DOI:** 10.2196/11579

**Published:** 2018-12-14

**Authors:** Stian Jessen, Jelena Mirkovic, Cornelia M Ruland

**Affiliations:** 1 Center for Shared Decision Making and Collaborative Care Research Division of Medicine Oslo University Hospital Oslo Norway; 2 Institute of Clinical Medicine Faculty of Medicine University of Oslo Oslo Norway

**Keywords:** gamification, gameful design, participatory design, co-design, mHealth, eHealth, self-management, chronic disease, patient requirements, patient participation, patient personal strengths

## Abstract

**Background:**

Gameful designs (gamification), using design pieces and concepts typically found in the world of games, is a promising approach to increase users’ engagement with, and adherence to, electronic health and mobile health (mHealth) tools. Even though both identifying and addressing users’ requirements and needs are important steps of designing information technology tools, little is known about the users’ requirements and preferences for gameful designs in the context of self-management of chronic conditions.

**Objective:**

This study aimed to present findings as well as the applied methods and design activities from a series of participatory design workshops with patients with chronic conditions, organized to generate and explore user needs, preferences, and ideas to the implementation of gameful designs in an mHealth self-management app.

**Methods:**

We conducted three sets of two consecutive co-design workshops with a total of 22 participants with chronic conditions. In the workshops, we applied participatory design methods to engage users in different activities such as design games, scenario making, prototyping, and sticky notes exercises. The workshops were filmed, and the participants’ interactions, written products, ideas, and suggestions were analyzed thematically.

**Results:**

During the workshops, the participants identified a wide range of requirements, concerns, and ideas for using the gameful elements in the design of an mHealth self-management app. Overall inputs on the design of the app concerned aspects such as providing a positive user experience by promoting collaboration and not visibly losing to someone or by designing all feedback in the app to be uplifting and positive. The participants provided both general inputs (regarding the degree of competitiveness, use of rewards, or possibilities for customization) and specific inputs (such as being able to customize the look of their avatars or by having rewards that can be exchanged for real-world goods in a gift shop). However, inputs also highlighted the importance of making tools that provide features that are meaningful and motivating on their own and do not only have to rely on gameful design features to make people use them.

**Conclusions:**

The main contribution in this study was users’ contextualized and richly described needs and requirements for gamefully designed mHealth tools for supporting chronic patients in self-management as well as the methods and techniques used to facilitate and support both the participant’s creativity and communication of ideas and inputs. The range, variety, and depth of the inputs from our participants also showed the appropriateness of our design approach and activities. These findings may be combined with literature and relevant theories to further inform in the selection and application of gameful designs in mHealth apps, or they can be used as a starting point for conducting more participatory workshops focused on co-designing gameful health apps.

## Introduction

### Background

As smartphones and other mobile devices become increasingly ubiquitous, more and more mobile health (mHealth) tools and apps to support people living with chronic illnesses in self-management are becoming available. Although mHealth tools show great promise for supporting people with chronic illnesses [1,2], their success is often contingent on them being used as intended by the designers [3-5]. To increase the likelihood of users adhering to tools or services, borrowing design traits and approaches from the world of games, typically called either gamification or gameful design, has become increasingly popular over the past decade [6,7]. As opposed to serious games, which are games developed with an added instructional or normative purpose or takeaway [8], gameful designs refer to the use of game design approaches and techniques in otherwise nongameful or nongamelike situations, services, or tools to increase the user’s enjoyment and motivation [8,9]. Typical applications and elements of gameful designs are, for example, competitions with either the app itself or other users, setting goals to accomplish, earning rewards such as points and badges, or having your own avatars [6,7,10,11]. Following Hamari et al’s definition of gamification [9], whether or not something is to be considered gamefully designed is not connected to what specific elements one uses, but how these are applied and, in the end, experienced by the users. In this study, we approach this similarly and define gameful designs as using design approaches and implementations from the world of games (in our otherwise nongame tool) to add a sense of playfulness and increase users’ overall enjoyment and engagement.

In the field of health and well-being, several gameful electronic health (eHealth) and mHealth tools for a range of different user groups and contexts have been created, such as smoking cessation [12], mental health [13], diabetes [14], medication adherence [15], and transitional care [16]. Still, some [10] also point to the limited number of gamified apps for health promotion in comparison with other fields such as education and business. Johnson et al [6], in a review of gamified tools for health and well-being, identified 19 empirical studies and reported that over half of the studies included had positive effects (59%), especially on behavioral outcomes such as physical activity, whereas the remaining 41% led to mixed or neutral outcomes. Even though many of these tools target changes in behavior, and there is an overlap with gameful design techniques and behavior change techniques [10,12], these are however not the same. If we consider again the definition of gameful designs as proposed by Hamari et al [9], this comes down to whether or not this is experienced as gameful by the users. Furthermore, and as reported by Johnson et al [6], gameful eHealth or mHealth tools also have the added possibility and potential to increase wellness and well-being by, for instance, providing pleasant designs and user experiences. The authors also found that the positive benefits of gamified mHealth tools are greater for users without preexisting motivation, compared with those already motivated to use the tools. Despite this, these findings should be interpreted with caution due to the relatively small number of studies currently published and their methodological limitations.

### Design Guidelines for Developing Gameful Designs

At present, there is a dearth of guidelines, principles, or frameworks for designing and developing gameful designs that are empirically validated or evidence based [17]. From reviewing design frameworks for gamification, Mora et al [18] identified 40 frameworks, of which only 1 is in the field of health care [19], and specifically concerns the design of rehabilitation systems. Here, the authors proposed a detailed workflow of the overall design process, in addition to outlining specific suggestions for activities with stakeholders. This framework has, to our knowledge, not been evaluated. “The Wheel of Sukr” [20] is another set of guidelines, concerning the design of gameful mHealth apps for the self-management of diabetes. Even though this has been evaluated through a questionnaire regarding its content, it has not yet been practically tested [21].

Discussing design frameworks in general, Deterding [17] argues that these mostly consist of selecting typical gameful elements or parts, such as points, badges, or competitions from a predefined list, and fitting these to your design or solution. This makes them generic, thus not taking into account the well-known fact that the experience of gameful designs is context-dependent [17]. As such, there is no one-size-fits-all solution [22,23], and the gamefully designed tools need to fit both the users and the context in which they will be used [7,17,24]. Finally, and as with the health care–specific frameworks mentioned above, evaluations of the frameworks themselves are rarely conducted.

Thus, we can surmise that currently there are no validated frameworks for designing eHealth or mHealth tools gamefully [18] or that the road to success for gamefully designed tools is not found by following formulaic approaches, but is rather highly dependent on both the users’ preferences and needs as well as the different contexts in which they are using the tools [17]. Even though there has been some investigation into people’s preferences of gameful designs [11], such findings are typically decontextualized, and knowledge about users’ specific needs and preferences for gameful and engaging designs is still mostly lacking [17].

### User Participation in Design Processes

Even though there is a lack of evaluated frameworks for gameful designs, most proposed guidelines or frameworks as well as literature concerning gameful and engaging eHealth or mHealth tools, emphasize the importance and value of keeping the design processes user-centric [17,18,24-26].

User-centered design processes focus on the needs, interests, and requirements of the users [27]. These processes can be placed in a continuum from expert-minded processes, which view users more as passive objects to be tapped for information, to participatory-minded processes, which include the users as co-designers [28]. *Participatory design* is firmly placed in the latter end of this continuum. More than just a design methodology, participatory design [29] takes the position that those whose future we are designing should not only have a voice but also a say in this process [30]. To achieve this, the approach is not only focused on the outcomes of design processes but also on the process itself, as it is a vehicle for enabling the co-designers’ meaningful participation. Supporting this, the following are among the core tenets of participatory design: (1) mutual learning between participants and designers to better understand each other and the real-life situations in which the designs will eventually be used, (2) equalization of power relations by providing a voice to those who often do not have one in the society, and (3) using and designing tools and techniques that enable and support the participatory practices necessary to allow the participants to communicate and collaborate in the design processes [31], for example, by enacting real-life situations, playing design games, or exploratory prototyping [32].

### Using Gamelike Design Activities in Co-Design Workshops

Previous literature has shown that framing design tasks in a gamelike manner can be well suited to support participants’ easier understanding of the activities at hand by giving them clear rules and game-pieces as well as promoting their collaboration and creativity during the co-design processes [33-35]. For instance, Nicholas et al [34] used a version of the game Snakes and Ladders as a basis for the participants’ design work. In another study, Brandt et al [36] describe a design game in which the players combine cards with pictures of situations, with cards presenting descriptive words to create stories about a persona. In general, design games typically share a randomized and open-ended nature, which can make it easier for the participants to create new and novel ideas [33,34].

A participatory design approach with gamelike activities should, therefore, be well suited for a design process that is not only sensitive to both the design goals of designers but also to the different preferences and needs of users as well as the different contexts in which the tool will be used. Even though there are published work related to using participatory approaches in the design of mHealth tools, rehabilitation games, and serious games [37-39], to our knowledge, little has been done in terms of co-designing gameful mHealth tools for people living with chronic illnesses.

### Study Aims

This study is part of a larger research project funded by the Research Council of Norway, “The Power of Personal Strengths—using gamification to support patients in chronic illness management.” The project’s goal is to design and develop a gameful mHealth tool to help people living with chronic illnesses (long-term physical and psychological health challenges) [40] identify and use their own personal strengths to manage their everyday challenges of living with chronic conditions. The concept of personal strengths has its foundation in positive psychology [41] and can be defined as people’s “positive traits reflected in thoughts, feelings, and behaviors” [42]. Simply put, a focus on strengths means emphasizing what is possible, valuable, and doable as opposed to only the deficit and problem focus one traditionally finds in medicine [43]. Previous research has shown that strength-based interventions among other can contribute positively to better moods and happiness [41] and increased general health and well-being [44]. Therefore, the main goal of the tool developed through this project is to, in a gameful and motivating fashion, help its users find and use their own personal strengths in overcoming their everyday challenges and how technology could help them do so.

Previously, we have reported on users’ and stakeholders’ needs and requirements of functionalities for the potential strength-based tool [45]. As the next step in our research project, this study describes co-design activities undertaken to inform and inspire the gameful and engaging designs of the self-management tool. Thus, the aims of this paper are twofold: (1) to explore new approaches for using participatory design methods in co-design sessions for designing a gameful mHealth intervention and (2) to identify user requirements and ideas for a gameful self-management for people living with chronic illnesses. As much of the existing publications concerning methods for gameful designs are terse in their descriptions of the creative design phases [24], this study’s presentation will provide the reader with a detailed description of the workshop’s activities, materials, and their rationale.

## Methods

In this paper, we report on the methods applied to, and the outcomes from a series of 2 connected participatory co-design workshops exploring users’ preferences and potential contexts of use for a gameful strength-based self-management tool for people with chronic illnesses.

### Participants

For the workshops, participants were recruited through 2 hospital educational centers in the northern and southern parts of Norway, as well as the youth council at a hospital in the Oslo region. The criteria for participation were being fluent in Norwegian, having a long-term health challenge, and being over the age of 16 years. This study was approved by the privacy ombudsman at Oslo University Hospital, and all participants, or their legal guardians, signed informed consent forms before taking part. The participants each received a gift card valued at Norwegian krone 250 (approximately US $30) as compensation for participating in each of the 2 workshops.

In total, 22 participants, 14 female and 8 males, aged between 17 and 64 years (mean age 35.5 years) took part in the workshops. Due to illness and scheduling, not all participants from the first workshop were able to participate in the second, and 3 new participants were recruited (see Table 1 for the participants’ background information and their distribution per workshop). All but 1 of the participants used a smartphone, and they on average rated themselves to 3.5 out of 5 on the question “how experienced are you with smartphones and or tablets.” Most used their phones for many more services other than just talking and messaging, and 13 of the 22 installed new apps at least monthly.

### Process: the Workshops

We conducted 2 connected co-design workshops with each of the 3 different participant groups during the summer and autumn of 2017. The workshops were held at the premises of each of the 3 participating institutions and facilitated by the first author (SJ). Working primarily as an observer, a researcher or research assistant from the project group supported the facilitator and took notes and photographs. The first workshop focused on exploring ideas on how to use gameful techniques and approaches in the design of mHealth-related technologies. The second workshop both continued from and built on the output of the former, with an emphasis on helping users find and mobilize their personal strengths and how the technology could be used to support this process in an engaging and meaningful manner.

The workshops were designed in line with the ideals of participatory design [31,32], and as such, a significant amount of time was spent on learning activities to enable the participants to meaningfully take part. As we are introducing the participants to many new and advanced concepts, the learning activities were designed and organized along the lines of modern science classes, by starting with what people already know on a topic, presenting new information and organizing the new and old information, before finally reflecting on and applying the new knowledge [46]. For our workshops, this meant beginning with learning focused activities and gradually transitioning toward more open and design-focused activities as the workshops progress. Furthermore, the main design tasks were themselves designed to be gamelike activities, as this has been shown to both engage and put participants in a creative and innovative state of mind [33-35]. To keep the participants both engaged and active for the entirety of the workshops and not overload them cognitively with new and demanding concepts, ideas, and tasks [47], the workshops were planned to last for around 2.5 hours. As we are working with people with chronic illnesses, keeping the workshops shorter would also make participating less of a burden to them. In addition, having 2 shorter workshops as opposed to 1 long workshop would allow the participants to reflect on the content and concepts between the 2 gatherings.

### Workshop 1

#### Introduction

The first workshop started with a round of introductions where everyone presented themselves before we gave a short presentation of our project and the reasoning behind it. We then explained the idea of using what makes games fun and motivating to create gameful designs. Through examples, we presented a range of games from different genres, fields, and contexts aiming to cover some games everyone liked (such as *Super Mario*, *Pitching Pennies*, *Crossword*, and *Monopoly*), stopping for further discussion when the participants had reflections or thoughts on what we were discussing.

#### Sticky Notes Exercise

**Figure 1:**
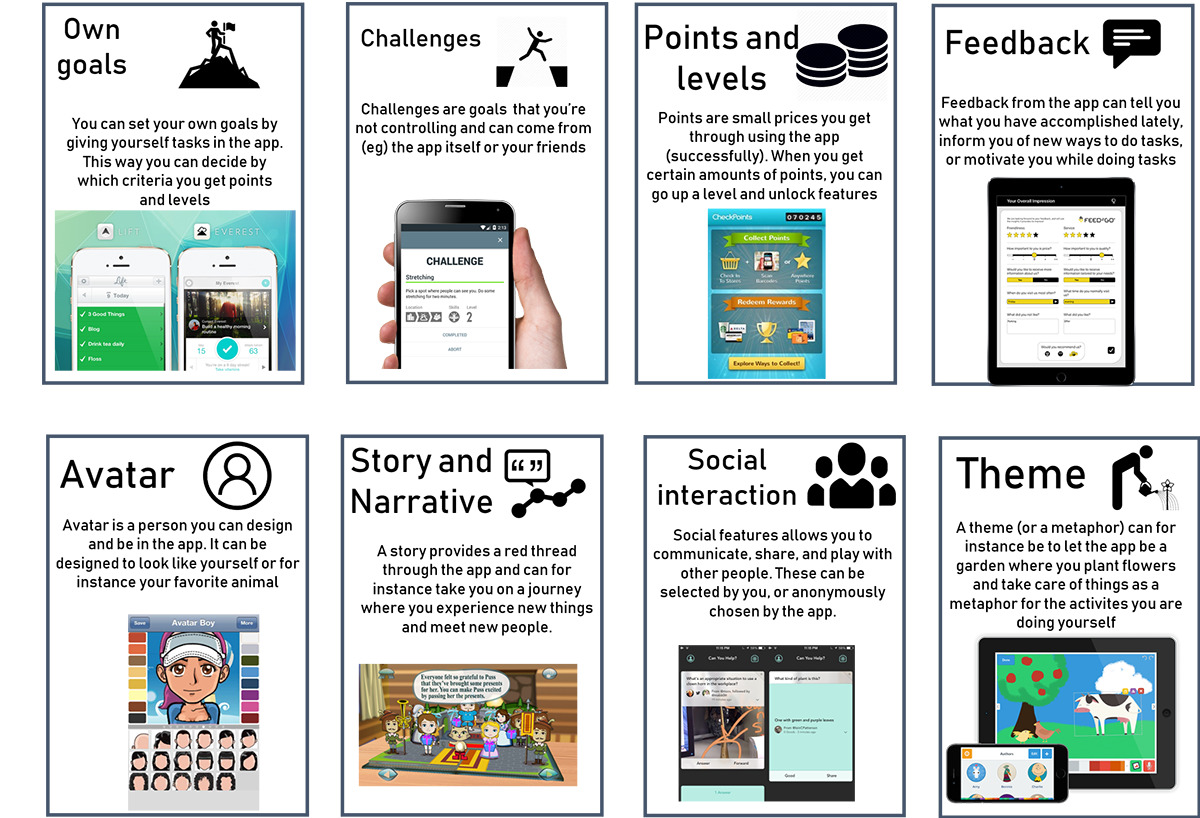
Presentation of game elements from workshop.

Next, we did a sticky notes activity where we asked the participants to note down on separate sticky notes the games they liked, why they liked them, and what feelings they evoked, and share this in group afterward. This task provided us with both the participants’ overall preferences of games and was the first step in thinking of games as a mix of smaller design pieces that together create the user experience. After discussing what the participants reported, we asked for games they did not like, why they did not like it, and how they would improve it. This latter activity gave the participants a taste of designing and putting together new ideas. Ending the first half of the workshop, we summarized what we had accomplished thus far and presented 8 different categories of game elements (see Figure 1 and Multimedia Appendix 1), based on Hamari et al [7], using games suggested during the sticky notes exercises as examples.

**Table 1:** Participant information.

Site	Workshop	Number of participants (n)	Mean age (range), years	Diagnosis (n)	How experienced are you with smartphones and tablets?	Highest completed education (n)
A	1	7	36 (21-58)	Attention deficit hyperactivity disorder (4); bipolar disorder (2); bipolar disorder and eating disorders (1)	3.7	Secondary school (5); university (2)
A	2	4	37 (21-58)	Attention deficit hyperactivity disorder (2); bipolar disorder (2)	3.7	Secondary school (3); university (1)
B	1	5	19 (17-21)	Chronic fatigue syndrome (1); Crohn disease (1); depression (1); chronic intestinal pseudo-obstruction, gastroparesis, spinal cord injury (1); not reported (1)	3.8	Secondary school (5)
B	2	4	20 (17-21)	Crohn disease (1); cerebral palsy (1); chronic regional pain syndrome (1); chronic intestinal pseudo-obstruction, gastroparesis, spinal cord injury (1)	4	Secondary school (4)
C	1	7	48 (27-64)	Chronic fatigue syndrome (2); spinal cord injury (2); fibromyalgia and posttraumatic stress disorder (1); hearing impairment (1); multiple sclerosis (1)	3.3	Primary school (1), secondary school (5); university (1)
C	2	6	50 (32-64)	Chronic fatigue syndrome (2); spinal cord injury (2); hearing impairment (1); multiple sclerosis (1)	3.0	Secondary school (5); university (1)

#### Design Game 1

**Figure 2:**
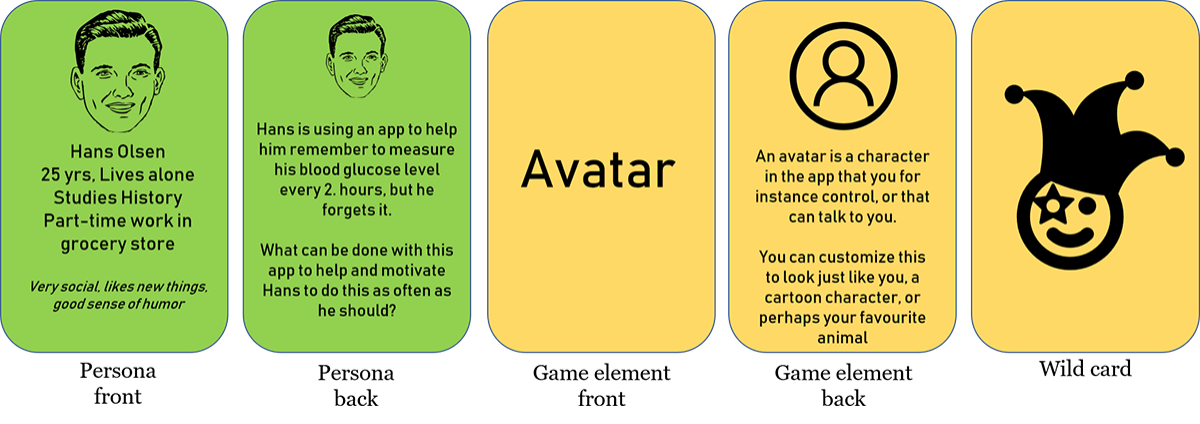
Cards from design game.

The second half of the workshop consisted of a 2-part design game. For the first part, we prepared a set of cards with (1) personas and design challenges and (2) game design elements (Figure 2). Personas are descriptive models or representations of unique users [48], and we created 3 personas that had generally known chronic illnesses with commonly known symptoms, challenges, and issues. The back of the persona cards featured a small design challenge specific for the persona. These challenges were based on tool functionality ideas identified in earlier work on the project [45]. All 3 personas are presented in full in Multimedia Appendix 2.

The game element cards, 8 in total, had the title of the element on the front, and a small descriptive icon and explanatory text with a few general examples of use on the back. We also provided a “wild card,” which could be whatever design element or approach the participants chose, to lessen the chance of the participants running out of ideas for their task and promote creativity.

**Figure 3:**
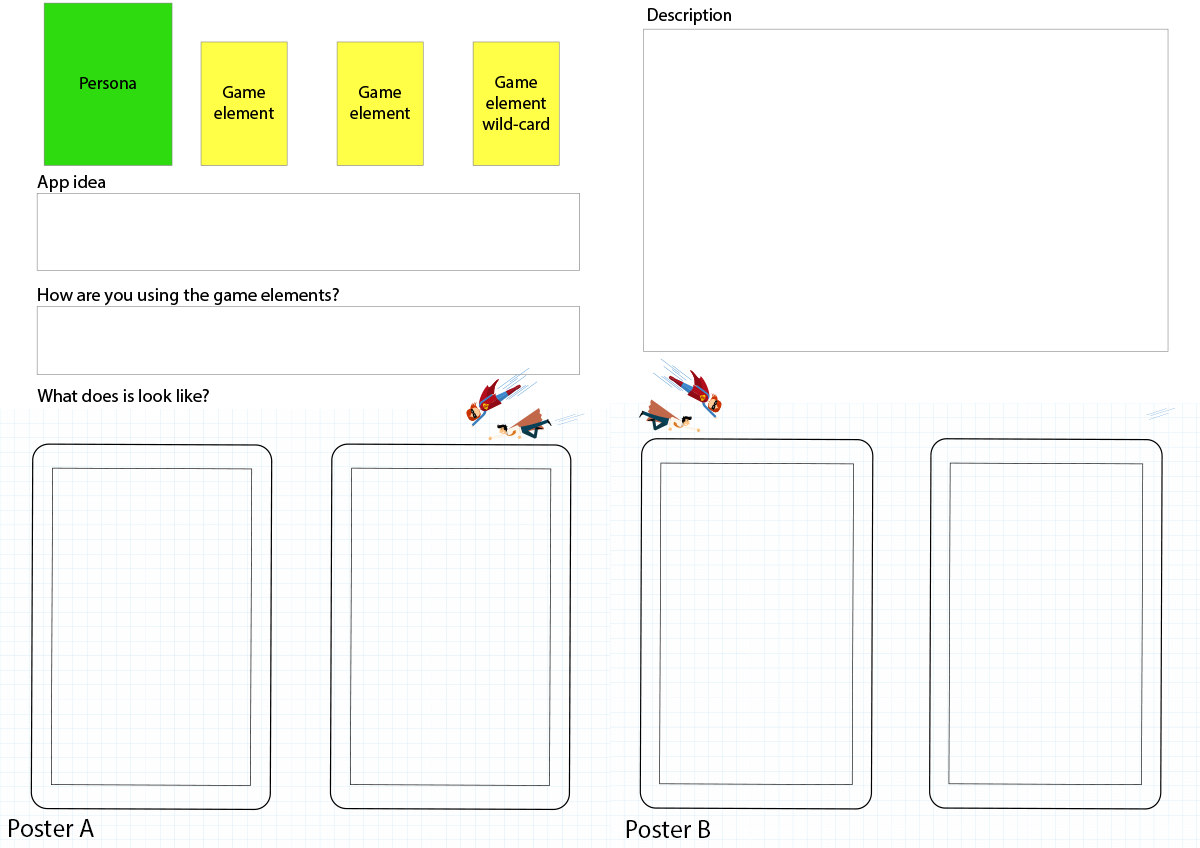
Posters for design proposals.

The participants were split into 2 smaller groups of 3 to 4 participants each, who worked independently of each other. Each group at random drew a card with a persona and a design challenge, 2 cards containing game elements, and got a game element wild card. The overall idea for this activity was to create an idea solving the challenge on the persona card by using the game elements cards. The facilitators were always available for discussion but did not partake in the groups’ work. To structure their work, the groups were given a poster to write down their ideas on (see poster A in Figure 3). After working for 20 min, the groups presented their ideas, the facilitator asked a few reflecting questions, and there was a short plenary discussion on the different game elements’ uses and ideas.

#### Design Game 2

**Figure 4:**
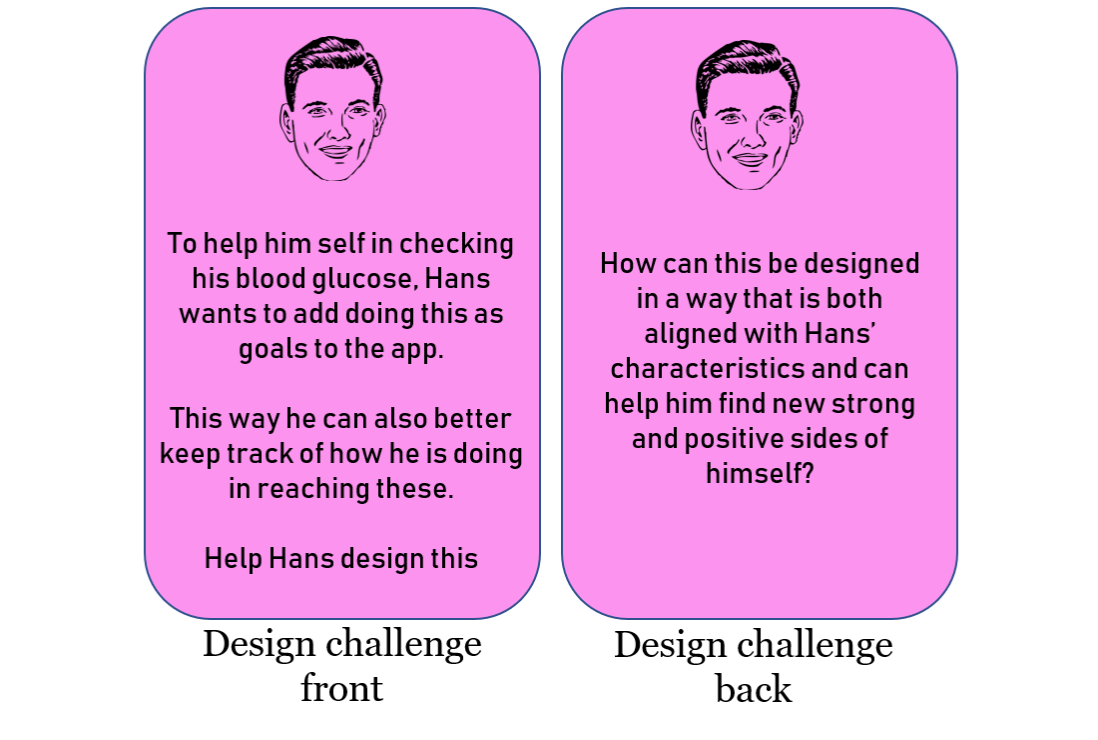
Cards for design game 2.

The second design game followed the same outline as the previous. The groups kept their persona but received a new design challenge card (see Figure 4) with a larger and more complex design challenge. As the groups now were familiar with how this design game was played, we removed the constraints of the groups having to use the design elements they had drawn, and they were free to design whatever and however they wanted. To write down and present their ideas, we provided an additional poster (poster B in Figure 3) with more room for describing and drawing their ideas and proposals. The groups got 30 min to work and then presented what they had come up with. As before, the facilitator then led a short plenary discussion on the different game elements’ uses and ideas.

#### End of Workshop 1

When the discussion had ended, the facilitator briefly summed up the workshop activities and thanked everyone for their participation. We gave the participants the gift cards and a small notebook and asked them to write down experiences of gameful designs they would have until the next workshop, where we would discuss these. Before leaving, we held a short plenary discussion for the participants to provide feedback on the workshop.

#### Planning and Design for Workshop 2

After the first round of workshop, the ideas and inputs from the participants were sorted and preliminarily analyzed. We then combined these design ideas and inputs with the previously identified and wanted functionality of our mHealth tool [45] and created a paper-based, low-fidelity prototype of our mHealth tool. This contained functionality for assessing your own strengths, setting goals, selecting strengths to help achieve your goals, and to collaborate with a friend. On the basis of the feedback and our own experiences, we also tried to make activities in the second workshop more concise to allow more time for group work. Finally, as it became clear that new people had to be recruited to the second workshop, we made the recap of the first workshop more detailed and comprehensive.

### Workshop 2

The second workshop was held with the same user groups at the same settings, approximately a month after the first. The main theme for this workshop was the design of the tool supporting the discovery and use of personal strengths*.* Maintaining the same overall structure as the first workshop, the second was built upon the results from the first and added the concept of personal strengths in the same manner as gameful designs in the first workshop.

#### Introduction

We started with a recap of our project’s aims, what gameful designs are, and our goal of designing the mHealth tool gamefully can make it more engaging to use. Thereafter, everyone had the opportunity to either present what they had written down in their notebooks or other thoughts and reflections they had since the last workshops regarding gameful designs. We then presented the concept of personal strengths and how basing care and self-management around your own strengths can improve quality of life and overall well-being. The participants then did a strengths identification exercise by selecting strengths items from a list of 30 personal strengths items that participants in previous studies have reported [43], such as “I am a social person” and “I like to try new things.” The participants volunteered to present their strengths and stories of situations in which they had used these. This was followed by a discussion concerning the exercise and reflections on the process. These tasks were performed to help the participants better understand the concept of strengths, experience how the strengths-identification process would look and feel, and create an overall positive atmosphere by reminding the participants of their own strengths.

#### Redesign Activity

**Figure 5:**
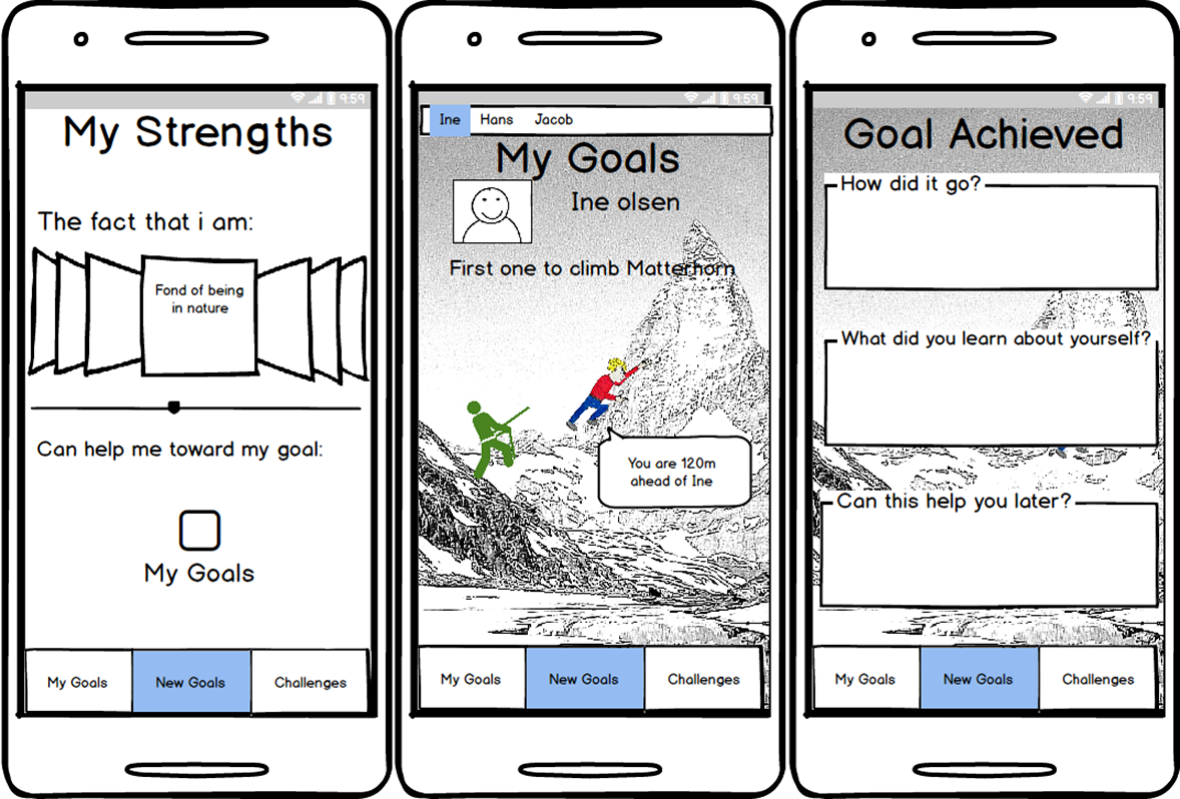
Paper prototypes for workshop 2.

We introduced the participants to the paper prototype of the app (Figure 5) and asked them to redesign it to make it better suited for finding and using more of their own personal strengths. The prototypes were printed on A4 size paper, clipped together to allow for easy reorganization, with ample room for notes and drawing. We also supplied *empty* wireframes for new drawings.

The participants worked for 20 min in groups and then presented their results. Thereafter, the facilitator led a short discussion, asking reflecting questions regarding the redesigned prototypes and the participants’ implementation of the strengths concept in these.

#### Design Game

**Figure 6:**
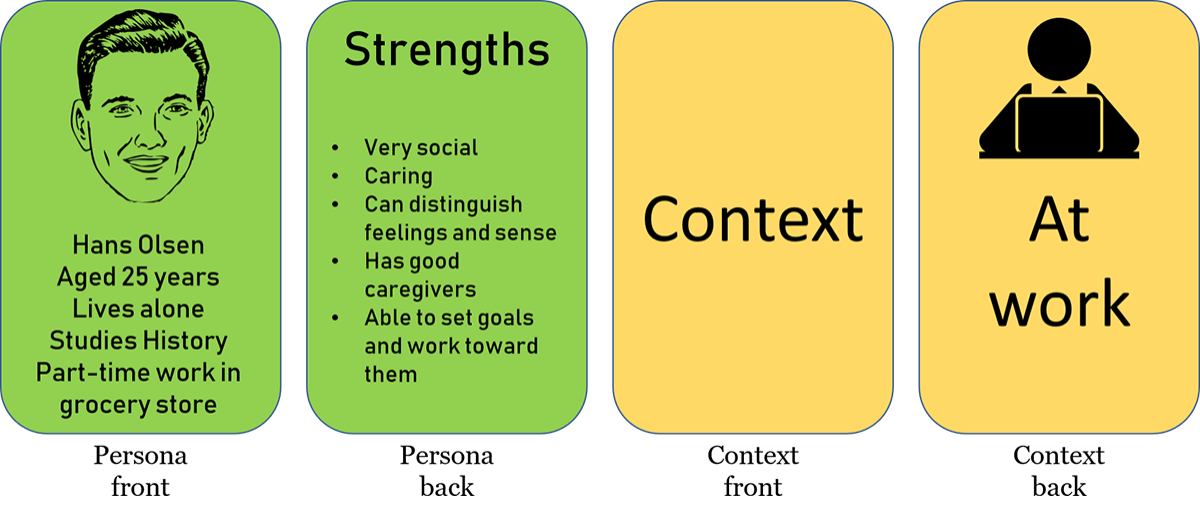
Cards for design game in second workshop.

For the final task, we used cards with the personas from the previous workshop. This time they were slightly rewritten, removing the design challenges and instead listing 5 of their strengths. We also provided cards (Figure 6) presenting a context in which the user would use the app (at home, at work, at school, with friends, at the doctor, and engaging in a recreational activity).

Each group drew a persona and a context card. The task was to describe how their persona would use their modified app in that context. After working for approximately 30 min, the groups presented their solutions as use scenarios. The discussion then continued on how the participants themselves could use such an app in their own context.

#### Ending Workshop 2

After the discussion, we summed up both workshops and presented the project’s future development plan. We then briefly discussed the participants’ experiences of the workshops before we thanked everyone for their participation, gave them gift cards, and ended the workshop.

### Data

The workshops were audio and video-recorded, totaling approximately 15 hours. Both the facilitator and the observer present took notes as well as photos during the workshops, and we collected all written materials created during the workshops. This provides us with 4 types of data (see Table 2). The recordings and written materials form the core data for our analysis, whereas the photos and notes add context and framing.

**Table 2:** Types of data.

Type	Description	Status
Audio/video recordings	Transcribed recordings of all 6 workshops, including individual groups	Core data
Written materials	Drawings, notes, sticky notes, and written ideas from the workshops	Core data
Photos	Photos taken during the workshops	Contextualizing data
Facilitators’ notes	Our own notes, written down during and after the workshops	Contextualizing data

### Analytical Approach

The data collected were analyzed thematically, guided by the 6 steps described by Braun et al [49]. The recordings were transcribed by the first author (SJ) and imported into QSR NVIVO 11 [50]. The first author made a first pass of coding, seeking inputs regarding use and experiences of gameful designs. New codes were generated as needed. To ensure that all data were coded with the same set of codes, the whole material was gone over a second time. When codes were related, overall categories were created, resulting in an hierarchy with 2 levels such as the category *Goals and Competitiveness,* which contains the 3 codes *Challenges from users or the app, Competition*, and *Setting goals/challenging yourself.* The codes and categories were discussed with the second author (JM), and inconsistencies or disagreements were discussed until an agreement was reached. The first author then made another pass to ensure that the entire corpus was coded from the updated codes and categories. The first and second author then together reviewed and agreed on a final set of categories and codes.

### Reflections on Reliability

In this study, reliability issues are addressed by following several of the strategies suggested by Creswell [51]. First, the workshops are held at 3 different sites and are conducted using both individual and collaborative methods and activities. Second, in the presentation of the results, we present both decontextualized extracts and examples of our coding, as well as 2 examples of the participant's whole app concepts. Third, as the second workshop builds upon the output of the first, it also functions as a form of member checking. Despite this, although we follow several suggested strategies for reliability in qualitative research, it is important to not view the output from the workshops as generalizable but as products of the situated activities.

## Results

### Overview

The coded data cover aspects of design such as gamelike features, look, feel, and overall user experience and were separated into 6 overall categories during analysis. In addition to the coded data, this section also presents 2 complete design concepts for self-management mHealth apps that 2 of the participant groups created during the design activities. These 2 concepts provide a macro view of the participants’ preferences, use, and combinations of different game elements.

### Points, Progress, and Rewards

Points, progress, and rewards or combinations of these were mentioned by all the groups. The ideas included getting recognition from the app for finishing tasks or being able to acquire points to unlock new functionality:

To get points when you have done something positive is kind of the easiest. You know, to get some recognition when you have done well.

Site C, WS1 male, 64, spinal cord injury

Some of the participants suggested having different types of points that are aligned with the users’ situation or context. For example, the user could obtain points for doing nothing or taking a break, as resting is important for many patients with chronic conditions such as fatigue. Some of the participants also suggested granting users control over what forms of rewards that will be used:

Maybe you can decide for yourself what you want as a reward then?

Site A WS1, woman 58, bipolar disorder

Points were popular with the participants, even going as far as one group suggesting awarding 100 points a day for each small task completed in the app. However, rewarding points were also discussed with trepidation, as pursuing more points can be both stressful and addictive:

But, points, doesn’t that stress you out when you should be relaxing with the app?

Site C WS1, male 47, hearing impairment and tinnitus

Exchanging points into rewards was an important topic of discussion. Some of the groups discussed linking rewards in the app to rewards in the real world, by having a gift shop at a hospital, where users can choose and exchange real-world rewards and presents for the points in the app (which is similar to how people donating blood in Norway are rewarded). Contrary to the idea of real-world rewards, several groups discussed having rewards in the virtual world, such as trophies:

If she wins something, it should be something she gets in the app, and not in the real world.

Site C WS1, female 37, fibromyalgia, posttraumatic stress disorder

### Goals, Challenges, and Competition

Most of the groups thought that users should be able to set their own goals and break these down to more manageable subgoals. Some suggested entering a goal when starting to use the app and then creating subgoals to achieve it.

Other ideas included tailoring the goals and challenges based on the users’ preferences situation. It was proposed that the app could do this automatically or by having someone working on the back end:

We think the app gives you challenges based on the goals you have set. Say you need to get better at feeling when your body needs rest, and then it [the app] will give you challenges that make you think about it.

Site B WS2, woman 21, chronic intestinal pseudo-obstruction, gastroparesis, spinal cord injury

Several ideas for increasing engagement revolved around the app enabling users to connect to and compete with others. However, participants also raised important concerns regarding the use of competitive elements in this context:

It can be tricky to let people compete or compare themselves against each other [referring to a prototype picture of two people climbing a mountain together]. This is fine if you’re alone, but to see others being better than you or you being poorer/worse can be hard if you’re lagging. There will always be someone at the back, and they may well be struggling the most.

Site 1 WS2, woman 29, attention deficit hyperactivity disorder

### Avatars and Feedback

Avatars were also proposed by many of the participants. An avatar could, for example, function as a tutor or guide to give the user feedback on activities or show and explain how to do certain exercises. Presenting information through avatars was discussed as potentially making it more meaningful:

I believe in that about avatars, having someone talk to you. Maybe not your parents, but perhaps yourself or a friend or something...Because I think having someone talk to you is more effective than just reading it on screen.

Site C WS1, male 64, spinal cord injury

Another use of avatars was to have it represent the user herself and for instance, visualize the users’ progression through the app or using the avatar to show how to do forms of exercises. With respect to the appearance of the avatar, it was suggested that users should be able to choose from a gallery of predefined avatars or create new ones on their own (for example, an avatar that can resemble the user or his/her favorite animal).

The participants also discussed the content of feedback users would receive, and the suggestions ranged from having the app delivering automatic predefined feedback to receiving it from peers who also use the app. When discussing feedback in general, many participants agreed that it should be mostly positive and productive, such as informing you of your accomplishments or providing help or guidance.

Concerning feedback in the form of notifications, the participants said they often view these as irritating and suggested that they should have a more meaningful purpose than to just remind users to use the app:

Not an app that gives lots of notifications like, you haven’t done this and that, but more like, Good, you did this! But not reminding of the negative, so that you get more energy out of it.

Site A WS1, woman 21, attention deficit hyperactivity disorder

### Social Features

Being able to share experiences, communicate, or collaborate with others are recognized by the participants as being powerful in terms of motivating and supporting an app’s users. Ideas included the ability to connect with others by communicating through chat rooms or forums. One idea was to enable others to cheer you on in your progress by one-directional messages of support. Having a button to easily ask others in similar situations for help is suggested by several groups. Some participants also suggested that a user could have one specific partner to collaborate with closely while using the app.

Keeping a positive focus was also important in this context. One group was so concerned with this that they suggested that users only should be able to send content from a set of predefined texts, icons, or emoticons to ensure all communication is of a positive nature and that there are no negative comments:

Being able to push and motivate. But it should not be that you can send negative messages to each other, so it could be an alternative to only be able to send pre-written messages like good, heart, stars and stuff.

Site B WS1, female 21, chronic intestinal pseudo-obstruction, gastroparesis, spinal cord injury

Several participants also discussed issues surrounding privacy, such as enabling the user to decide who to share what with or whether to share things at all:

Cause’, if it is private it is much easier to be totally honest. Sometimes you have strengths you might not want to share with anyone else.

Site B WS1, female 17, did not report diagnosis

### Themes, Stories, and Narratives

Several groups suggested overall themes such as designing the app as a journey to exploring countries or continents and gradually unlocking new locations and activities. Other themes were a 400-meter sports-track with hurdles and other obstacles representing smaller goals or challenges. One group suggested climbing mountains as a theme that can both represent the users’ goal and progression. Similarly, another group proposed having a theme of being in nature (which is a very common recreational activity in Norway), for example, moving through a forest in an unfolding story as a narrative:

It could be that you walk into a forest where something exciting is going to happen, maybe a story, and for each morning you go further in there.

Site A WS1 woman 58, bipolar disorder

Combining the idea of a narrative with the rewards in the app, another group suggested theming the app as a mystery story with rewards that unlock new chapters.

Regarding the personalization and fit of themes, one group also suggested that users should be able to choose between different themes after their own liking:

The app could be related to something you like. For instance, he likes working on cars, so perhaps instead of climbing mountains he gets a car in pieces he has to assemble.

Site A WS2, male 40, attention deficit hyperactivity disorder

### Engaging Visuals, Sounds, and Texts

The participants’ suggestions ranged from very specific needs (for example, sizes or look of specific buttons), to the general need and guiding principles for the app’s “cool look.” For instance, one group suggested that the home screen could be a “boasting wall,” showing off the users’ successes and strengths. Focusing on the positive, using images of times of success and happiness was mentioned by several groups as being powerful reminders and positive boosts during negative periods.

Other ideas included using a scrolling wheel instead of drop-down lists to adjust dates and times or having variation in the content and notification provided by the app. Interestingly, several groups also discussed the need for the app to be something new and innovative, not just copying features of other tools or services:

It’s starting to be very similar to Facebook now, and it should not be that similar to other apps.

Site A WS2, male 40, attention deficit hyperactivity disorder

Although engaging design elements were proposed and described, the participants were very cautious about how these could affect the apps’ usability and intuitiveness. For example, one group said the app should not have too many different buttons and menus, as this could be confusing:

It should be simple, and with easy and quick access. If there are people around 60 and 70…they might not understand everything, and may not find out how to use it.

Site A WS2, male 21, attention deficit hyperactivity disorder

Several groups also added to the immersion of the themes by suggesting sounds or music in the app that are topically proper, such as sounds of the forest or a windy mountain. Regarding textual content, the participants mostly agreed that the design elements should be presented as audio or video rather than just text. Some also discussed the different experiences that text and video can provide:

Yea, and if it’s video then you get more the feeling that it's talking directly to you then if you’re reading it.

Site B WS2, male 17, cerebral palsy

### Two Design Concepts

This section presents summaries of 2 app concepts that were generated by the participants during the workshops. These serve both to highlight the complexity of the systems the participants created during the workshops and provide a macro level and more contextualized view on how the participants assembled the various microlevel, design elements.

#### Idea 1: A Journey Toward Mindfulness (Site C Workshop 1)

The goal of this app concept is to help the user perform mindfulness exercises. The app uses a journey to different parts of the world as a metaphor. The user can travel to different areas and countries with levels or stages that can be gradually unlocked. Each stage contains new exercises especially themed and tailored to the area. For instance, with India as the destination, the app uses Indian-themed symbols, sound effects, music, and provides mindfulness and yoga exercises based on the given region. When the user goes to another place, the content is themed for the new location. Elaborating on this during the second design game, the participants suggested adding a feature that lets a person from the users’ personal network, such as a partner or a parent, provide support with encouraging messages through the journey. The app should also allow the user to add pictures of happy times and situations that can be used both as rewards and reminders, for instance in periods when one is feeling depressed. The participants also suggested a feature that enables the user to communicate with others in the same situation by sharing new places discovered on your journey in the app as well as documenting and sharing physical places that provide meaning, joy, or relaxation in their real life.

**Figure 7:**
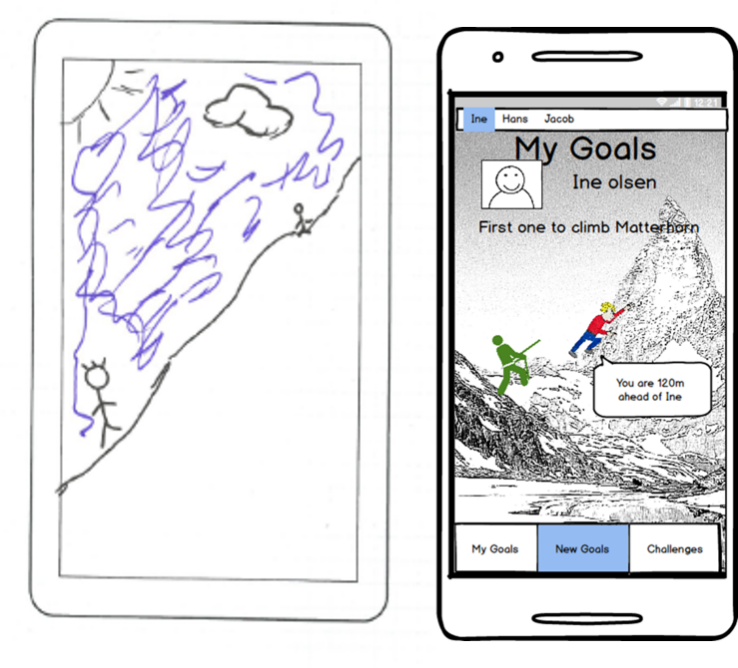
Participant drawing from workshop 1, and refined prototype wireframe for workshop 2.

#### Idea 2: Climbing Mountains of Challenges (Site B Workshop 1)

The other large design concept developed by the participants involved helping a user reach their goals. The apps’ main metaphor is mountain climbing (see drawing in Figure 7). The app allows a user to design his or her own avatar and the movement of this avatar across the mountain serves as a visualization of the users’ movement toward his or her goals. The avatar can also show and explain various tasks or exercises the users will encounter along their journey up the mountain. These mountains are modeled on real mountains, and their height is relative to the users’ progression in the app. This app was also designed to provide the users with sounds of nature to add to the immersion. Users also have the option to communicate with others and add them to their app and climb together. The app would then visualize how both users are scaling the mountain in relation to each other.

## Discussion

### Participatory Methods

In this study, we organized a series of participatory design workshops with people living with chronic illnesses to jointly explore preferences, requirements, and ideas for gameful mHealth tools. The results of the study showed that engaging the participants with gamelike activities supported them to be collaborative, effective, and creative, especially by applying activities that set particular rules to their interaction (such as the rules of the game itself and the restriction of design elements). In addition, this approach provided the participants with a direction for their exploration of new ideas through, for example, the personas with their connected design challenges and the game elements cards. These findings are in line with previous studies that explore using gamelike participatory activities in design processes [33-35].

Using card-based design tools and activities are common to both design in general [39,52] and to game and gameful designs [17,53]. Even though it is important to not only focus on the application of specific elements but also adapting them specifically to the target users and their context, this is often overlooked and not addressed properly in design processes [17]. In our study, we addressed this by the following: (1) having the persona card provide a clear context for both the use of the future tool and the user and (2) defining design challenges in the context of system functionality and requirements identified by users in earlier phases of the project. The game design element cards were also designed to be broad, leaving it to the participants to decide the specific interpretation and use of the elements. Thus, the participants were staying within the specific context and scope of our project while having room to freely ideate and be creative. Interestingly, it seems that the participants used the design cards more as what is termed *inspiration cards* [54], which often carry images, words, or short statements that are used as a point of departure for further discussion and exploration. Our participants used these cards as a starting point in the process of discovering possible design ideas and solutions. For instance, when some of the groups discussed using rewards in their idea, they not only discussed the reward as any generic reward but also how it can be used to (1) provide social activity by being a free dinner out with friends or (2) give positive boosts to the user by showing photos uploaded by either the user or their partner. As such, the participants used the game element cards freely and creatively and thus somewhat opposite to their often very specific and more prescriptive uses in design activities [17].

However, we also experienced that facilitating a process that not only supports both openness for the participants to be creative and innovate but also provides rules to keep their work within the boundaries of our project can be challenging. One example of how we addressed this issue is by limiting the number of game element cards the participants drew during the activities but also giving them a wild card that provided the freedom to use any gamelike approach they liked. In this manner, the participants were simultaneously provided with (1) a gamelike experience where they received different game elements by chance, (2) support in choosing the design concepts to use as a starting point, and (3) the possibility to freely explore features and elements other than those they had randomly drawn. The 3 different groups of participants in this study all had a different character and behavior, and although it is important to find a good balance of openness and rules during the planning of the design activities, some adjustments still had to be made on the fly during the workshops.

User involvement in the design process can play an integral part in *widening the design space* by contributing choices and ideas to the design project, stemming from their own imaginations of future uses of such tools [55]. The participants in our study came up with a great range of ideas and design proposals, showcasing a collective creativity that greatly adds to and extends that of the professional designers, developers, and researchers in our project team. Some of these ideas include having a gift shop in which you can exchange virtual points for real-world gifts, being able to add own photos that can be used as rewards and positive reminders, or the button you could push to easily get in contact with people in similar situations. However, as a wide range of ideas and suggestions were reported and discussed by the participants during the workshops, some of these are at times at odds with each other, such as the ideas of having competitions in the app and the wish to not visibly lose to someone else. Some ideas are also counter to evidence and design principles. For example, one group suggested awarding 100 points for each of the 8 completed small tasks during a day, for a total of 800 points as a score for completion. Although rewarding points are one of the more popular gameful design elements [6,7], it is also known that one does not engage users more by inflating the rewards by as suggested, giving 100 instead of a single point [17]. Therefore, even though involving users is both important and valuable, one must still make sure design decisions are made in accordance with relevant literature and evidence concerning both the design and content of the tool that is being made.

Overall, we can conclude from the vast variety of user inputs that the workshops were successful in generating new and creative concepts and ideas for mHealth tools. It served as a vehicle for the participants to gain new knowledge from this domain and communicate their requirements and needs. However, giving the participants the freedom to interpret their own tasks also allowed them to veer in directions that can be unproductive (as with the example of awarding 100 points at a time) or impossible to implement. At the same time, it is hard to correct participants when they veer outside our topic without seeming critical or negative, and in these few cases, we mostly let them continue. Despite this, even though such diversions may be unproductive in terms of creating design ideas, they still expand the knowledge base and overall output of the design activities. One thing that did not work as intended was the notebooks given to the participants after workshop 1. Many had misplaced or simply forgotten about these between the 2 workshops, and in future studies, we will consider either using text messages or social media to remind the participants of such tasks. The participants and the facilitators alike found the workshops to be both productive and enjoyable. In fact, when getting feedback at the end of the first workshop, all 3 groups wanted to spend more time on the next workshop.

### Design Ideas and Requirements

As presented in the Results section, the workshops yielded a range of ideas and requirements for designing mHealth tools gamefully. For example, the use of metaphors, which is a well-known valuable design approach to increase motivation and use of mHealth tools [25,56], was frequently proposed by participants in the study. As exemplified by the 2 design concepts, “Journey towards mindfulness” and “Climbing mountains of challenge,” the participants confirmed that the use of an overarching theme or metaphor can be a suitable approach to designing mHealth tools. However, we also noticed that many of the proposed metaphors are culturally and context specific, which limits their overall generalizability. For example, hiking mountains and being outdoors in nature is a popular recreational activity in Norway but possibly not equally appealing for people living without easy access to nature. Similarly, chapters of a story as rewards could be engaging only for users interested in the story. Therefore, we can conclude that although metaphors can be a powerful and engaging design element, they need to be fitting to the target users, and one way to ensure this is, as also suggested, to allow users to choose between several themes or styles of metaphors.

Social functionalities are commonly employed in mHealth tools to provide collaboration or communication between users [3,6,57]. Such features were also often suggested by the participants as a powerful way for both getting support from, and being motivated through interaction with others. However, it is important for designers to be careful about how they implement such features for sensitive groups, such as people living with chronic illnesses [13,58,59]. This was discussed on multiple occasions by the participants, and they voiced both opportunities and concerns. On the beneficial side, having others to communicate with can be a great source of inspiration and support during hard times. Being part of a group or community with others in similar situations or with the same diagnosis was also mentioned as a good way for obtaining advice. This is in line with, amongst others, findings from research on the *Patients like me* network [60] that showed users advising and supporting others in similar situations based on their own personal experiences. On the other hand, being able to compete or compare one’s own progression with other users of the app was also mentioned as being detrimental to motivation and joy in general. This is similar to what the study by Chen Y et al [61] reported, which stated that competition between people with different abilities and performance could be experienced as demotivating. During the workshops, the participants also often touched upon the changing shape or mood people living with chronic illness experience and highlighted the importance of taking this into consideration when designing features for mHealth tools that, for instance, offer social comparison or competition with others.

As mentioned, awarding points and rewards are among the most commonly used gameful design elements [6,7], and were also one of the more popular design features suggested by the participants. Besides awarding points in the app, most groups also discussed the possibility of rewards outside of the app. This is in line with Nicholson [23] who argued that rewarding not only virtually but also with something tangible can be experienced as more meaningful by the users. In addition, approaches such as pursuing rewards have also been reported as unfit in some of the health-related contexts such as mental health and mindfulness [13]. This is also reported by the participants, who often voice concerns regarding using designs that rely heavily on collecting points and trophies or rewarding use with streaks. Thus, we can conclude that combining both external rewards such as points in the app with more personal and intrinsic rewards (such as a real-world gift of your choice or going to dinner with your friends) can be a promising approach for providing rewards as part of gameful designs in this context.

Previous research has shown that personalization and allowing users to customize their own gameful tools might be a way of alleviating the issues of one-size-fits-all designs [17]. Personalization of the mHealth services by, for example, tailoring messages or allowing the users to customize the appearance or behavior of the service, can be an important mediator for user satisfaction and enjoyment of services [25]. In addition, personalization can broaden the reach of metaphors, social features, competitive elements, and rewards by having the users adapt these to their own preferences [25], something also suggested by the participants. In terms of designing for positive and more engaging user experiences, the participants proposed a range of relevant ideas and solutions, such as adding one’s own music, designing one’s own avatar, or setting one’s own goals.

### Every Stone Is a Keystone

For gameful designs in general, the results gained from this series of workshops highlight the complexity of both designing and experiencing gameful tools. We saw that the participants used the different design elements in highly interconnected ways and sometimes had them build on each other to form overall concepts or ideas for a tool. One example is the idea of climbing mountains with a friend. The overall idea was providing a sense of a competition (2 users compete for reaching the top of the mountain), but it is also designed as a social feature (you compete with someone) and a way of monitoring progress (climbing the mountain visualizes both users’ progress). This highlights how the experience of gameful designs is not a product of individual elements such as trophies or avatar but rather a product of the interaction with the gameful tool or service as a whole—something that is also often discussed in existing literature [9,62,63].

For both this and future work on co-designing gameful tools and apps, it is thus important to be considerate when combining pieces from different proposals or ideas coming from co-designers, as when you combine pieces from different ideas, you also create new and different overall user experience. Including end users throughout the design process and being open to their needs, requirements, and inputs are therefore important for ensuring that users find the final tools both meaningful and valuable [25].

### Strengths and Limitations

Both the methods used and the findings from this study can serve as a backing for future work and research on creating gameful designs for and with people with chronic illnesses. However, due to the explorative nature of this study, any generalizations as to what gameful approaches or designs people living with chronic illnesses enjoy or want is neither possible nor intended. Yet, we can conclude that the chosen methods worked well with 3 different groups and may be applicable to others as well. Furthermore, the heterogeneity of the participant group may be considered a limitation as the size of any participant subgroup (be it group by age, gender, or illness) is small. Nonetheless, this also allowed us to get feedback and input from many different viewpoints. It should be noted that the participants were all recruited from active users of the hospital youth council or education centers and most were also active in patient organizations. As such, this participant group is possibly somewhat biased in that they are resourceful and able to manage their life well with a chronic illness and not necessarily representative of the overall population of chronic patients. However, using *empowered* users is common in this phase of design projects, as they typically have more experience in addressing the existing problems and may have more reflective thoughts about their situation. Moreover, in a study like this, recruiting a convenience sample is often necessary to find participants willing to take part. Finally, the gender balance among the participants is skewed with 14 women and 8 men, which may have influenced the ideas from the workshops.

### Conclusions

In this study, we used participatory design methods to jointly explore, together with people living with chronic illnesses, their preferences, requirements, and ideas for designing gameful and engaging mHealth tools. Through gamelike design activities, the participants were both engaged, creative, and voiced a wide range of ideas and requirements. Much of the reported input and ideas are in line with previous research and provide important contextualization and nuance to these design choices from the users’ perspective, although we cannot generalize from the findings. As such, both the participants’ needs and requirements as well as the applied methods and activities add to a growing body of literature in the field of designing mHealth and eHealth tools in engaging ways by implementing gameful design features.

Both the methods used in and the results from this study could be used as a starting point for future studies exploring requirements of gameful designs in depth with other user groups, and we invite others to both further develop, adapt, and build on these activities for their contexts.

## Appendix

Multimedia Appendix 1 Game elements.

Multimedia Appendix 2 Personas.

## Abbreviations

eHealth: electronic health
mHealth: mobile health
