# Supplementary material for: Creating Gameful Design in mHealth: A Participatory Co-Design Approach
Source: JMIR Mhealth Uhealth. 2018 Dec 14;6(12):e11579. doi: 10.2196/11579 (PMC6315237; doi:10.2196/11579)
Supplement: Multimedia Appendix 1 [file mhealth_v6i12e11579_app1.pdf]

2. Table of Game elements

Game element	Description
Own goals	Goals you can set for yourself
Challenges	Goals set by friends or the app itself
Feedback	The app can tell you how you're doing, or cheer you on
Points & trophies	You can get points for doing activities or tasks or trophies for fulfilling a series of tasks. You can also use a number of points as a threshold for various levels in the game
Story	The app can be in the way of a story or adventure that you embark on, thus pulling you through it
Theme	A theme can be making the app, or visuals in it, be metaphors for other things we know.
Social features	Talking with friends, or the opportunity to challenge them.
Avatar	An avatar can be a figure/person in the app that you can design to look like you or whatever you like.
