# Supplementary material for: Creating Gameful Design in mHealth: A Participatory Co-Design Approach
Source: JMIR Mhealth Uhealth. 2018 Dec 14;6(12):e11579. doi: 10.2196/11579 (PMC6315237; doi:10.2196/11579)
Supplement: Multimedia Appendix 2 [file mhealth_v6i12e11579_app2.pdf]

Appendix

1. The three personas

	**Hans**	**Eva**	**Miriam**
**Info**	• 25 years, Lives by himself<br>• Studies history, work part-time in a shop<br>• Social, likes new things, good sense of humor	• 18 years, lives with parents<br>• Last year in high school, works part-time in pharmacy, used to play handball<br>• Caring, targeted, has good control of own limits	• 29 years, lives with Boyfriend<br>• Works as a teacher at a primary school<br>• Creative, enjoys being in nature, optimistic
**Illness**	Diabetes	Chronic fatigue	Chronic pain
**Challenge 1**	Make an app for checking blood glucose more engaging.	Make a tool for logging, tracking, and prioritizing activities more engaging.	Make an app for doing meditation exercises more engaging.
**Challenge 2**	Expand the app to include functionality for setting goals and keeping tracks of these.	Expand the app and add functionality for looking back on past activities and identifying what strengths and characteristics helped her achieve this.	Expand the app and add functionalities for looking back on past successes and achievements to lift her spirits when Miriam is feeling down.
